# Fellow-Workers and the Roll of Honour

**Published:** 1915-02-13

**Authors:** 


### ROUND THE WORLD.

[The Editor invites Early Information and Contributions Marked "Fellow-Workers."

### Fellow-Workers and the Roll of Honour.

The Liverpool Merchants' Hospital, which has been equipped as a mobile institution for rendering first-aid on the field of battle, will have for its senior physician and senior surgeon Lieutenant-Colonel Nathan Raw and Major T. C. Litler Jones respectively. These officers will work in connection with a military chief, and have under them a staff of about a hundred nurses and orderlies.

Lieutenant-Colonel Nathan Raw is a Liverpool physician who has done a great deal of work in connection with the treatment of tuberculosis, having made important observations both in regard to the open-air system and the tuberculin method. He is an M.D. Durh., F.R.C.S. Edin., and M.R.C.P. Lond., and holds the appointments of visiting medical superintendent to the Mill Road Infirmary at Liverpool, visiting physician to the Haydock Lodge Asylum, and physician to the Sanatorium for Consumption at Heswall. Colonel Nathan Raw, who is also chairman of the After-care Committee of the National Association for the Prevention of Consumption, and a British member of the International Committee for the Prevention of Consumption, has published numerous works and papers in connection with his special subject.

In private life Major Thomas Caldwell Litler Jones is a Liverpool operating surgeon A "Bart.'s" man, he became an F.R.C.S. Eng. in 1902, and is now honorary surgeon to the Liverpool Royal Infirmary and lecturer on clinical surgery at Liverpool University. In the South African War he served with the Field Force as civil surgeon, and has also done work as surgeon in the Royal Naval Volunteer Reserve. Major T. C. L. Jones has held the appointments of house surgeon at St. Bartholomew's Hospital and special plague medical officer in Bombay.

Sir John F. H. Broadbent, Bt., who has been appointed physician to the King George Hospital, is physician to St. Mary's Hospital, Paddington, assistant physician to the London Fever Hospital, and was at one time physician to the Hampstead General Hospital. He is also dean of St. Mary's Hospital Medical School and joint lecturer in medicine there. An M.D., B.Ch. Oxon., F.R.C.P. Lond., Sir John Broadbent, who is the author of various publications on diseases of the heart and circulation, succeeded in 1907 to the baronetcy conferred on his father, the late Sir William Broadbent, whose name is one of the most distinguished in British medicine.

Dr. J. A. Ormerod, M.D. Oxon., F.R.C.P. Lond., who is also one of the physicians at the King George Hospital, has been registrar to the Royal College of Physicians, London, for the past six years. An eminent consultant, he is consulting physician to St. Bartholomew's Hospital and the National Hospital for Paralysis and Epilepsy in Queen Square, and his work in the cause of scientific medicine has been referred to in more detail in these notes on a previous occasion.

Captain Arthur Claypon Horner Gray, who has gone abroad in charge of the Princess Christian Motor Bacteriological Laboratory, is a Guy's man who qualified M.R.C.S. Eng., L.R.C.P. Lond., in 1901, after which he held the posts of resident obstetric physician and house surgeon at his hospital. Taking the M.B. Lond. and entering the R.A.M.C., he quickly distinguished himself, winning the Parkes medal and Herbert prize at the Royal Army Medical College. Soon after, he was appointed a member of the Royal Society's Sleeping Sickness Commission in Uganda, on which he worked two years (1904-1906), his observations and conclusions having been recorded in an important report he drew up in collaboration with Major E. D. W. Greig and the late Lieutenant F. M. G. Tulloch, who, it will be remembered, became fatally infected with the disease being investigated. The laboratory is built on a powerful Clement-Talbot chassis, and so arranged that the main compartment is 6 ft. 6 in. long and 6 ft. wide, being thoroughly furnished with every essential for advanced bacteriological work.

Professor Charles J. Martin, who has been placed in charge of the bacteriological and pathological department of the same special institution for the care of the wounded, is director of the Lister Institute of Preventive Medicine. A student of St. Thomas's, where he early distinguished himself by taking the exhibition and gold medal at the Intermediate M.B. Lond., and a university scholarship in physiology at the B.Sc. Lond., he qualified in 1889 and was made an F.R.C.P. Lond. the year before last. Dr. Martin's valuable researches have brought him many academic distinctions, including the F.R.S. Lond. and the D.Sc. degrees of more than one university. Amongst his present appointments may be mentioned those of professor of pathology at London University, and honorary pathologist to the Victoria Hospital for Children at Chelsea; whilst he is also a member of the Advisory Committee for the Investigation of Plague in India and of the Royal Society's Committee on Tropical Medicine.

The routine work of this department will be in the hands of Dr. John C. G. Ledingham, Dr. W. J. Penfold, and Dr. Percival Hartley, all of whom have had much experience of modern methods of pathological research and bacteriological therapeutics.

Dr. John Charles Grant Ledingham is chief bacteriologist to the Lister Institute and reader of bacteriology at the University of London. An M.A., M.B., Ch.B., D.Sc. of Aberdeen University, all of which degrees he took with honours, Dr. Ledingham found himself attracted by the more highly scientific side of medicine, and carried out research work as Carnegie Research Fellow in pathology. His investigations have included important observations on phagocytosis and other phenomena of blood reaction.

Dr. William James Penfold is assistant bacteriologist to the Lister Institute, lecturer on bacteriology at the Royal Dental Hospital, and pathologist to the Royal Victoria Hospital for Children. An M.B., C.M. Edin. and D.P.H. Durh., he also studied at the London Hospital, where he took the diploma of the conjoint board in 1909. A lecturer on advanced medical subjects at London University and a British Medical Association research scholar, Dr. Penfold has carried out much interesting work on bacteriological variations and the relation of microbic activity to the production of fever.

